# Turnover Rates of Intermediate Sulfur Species (${\text{S}}_{\text{x}}^{2-}$, S^0^, S_2_${\text{O}}_{3}^{2-}$, S_4_${\text{O}}_{6}^{2-}$, ${\text{SO}}_{3}^{2-}$) in Anoxic Freshwater and Sediments

**DOI:** 10.3389/fmicb.2017.02551

**Published:** 2017-12-21

**Authors:** Alyssa J. Findlay, Alexey Kamyshny

**Affiliations:** Department of Geological and Environmental Sciences, Ben-Gurion University of the Negev, Beer Sheva, Israel

**Keywords:** sulfur, sulfide oxidation, intermediate sulfur species, sulfur biogeochemistry, thiosulfate

## Abstract

The microbial reduction of sulfate to sulfide coupled to organic matter oxidation followed by the transformation of sulfide back to sulfate drives a dynamic sulfur cycle in a variety of environments. The oxidative part of the sulfur cycle in particular is difficult to constrain because the eight electron oxidation of sulfide to sulfate occurs stepwise via a suite of biological and chemical pathways and produces a wide variety of intermediates (${\text{S}}_{\text{x}}^{2-}$, S^0^, S_2_${\text{O}}_{3}^{2-}$, S_4_${\text{O}}_{6}^{2-}$, and ${\text{SO}}_{3}^{2-}$), which may in turn be oxidized, reduced or disproportionated. Although the potential processes affecting these intermediates are well-known from microbial culture and geochemical studies, their significance and rates in the environment are not well constrained. In the study presented here, time-course concentration measurements of intermediate sulfur species were made in amended freshwater water column and sediment incubation experiments in order to constrain consumption rates and processes. In sediment incubations, consumption rates were ${\text{S}}_{\text{colloidal}}^{0}>$ ${\text{S}}_{\text{x}}^{2-}>$ ${\text{SO}}_{3}^{2-}\approx$ S_4_${\text{O}}_{6}^{2-}>$ S_2_${\text{O}}_{3}^{2-}$, which is consistent with previous measurements of ${\text{SO}}_{3}^{2-}$, S_4_${\text{O}}_{6}^{2-}$, and S_2_${\text{O}}_{3}^{2-}$ consumption rates in marine sediments. In water column incubations, however, the relative reactivity was ${\text{S}}_{\text{colloidal}}^{0}>$ ${\text{SO}}_{3}^{2-}>$ ${\text{S}}_{\text{x}}^{2-}>$ S_2_${\text{O}}_{3}^{2-}>$ S_4_${\text{O}}_{6}^{2-}$. Consumption of thiosulfate, tetrathionate and sulfite was primarily biological, whereas it was not possible to distinguish between abiotic and biological polysulfide consumption in either aqueous or sediment incubations. ${\text{S}}_{\text{colloidal}}^{0}$ consumption in water column experiments was biologically mediated, however, rapid sedimentary consumption was likely due to reactions with iron minerals. These experiments provide important constraints on the biogeochemical reactivity of intermediate sulfur species and give further insight into the diversity of biological and geochemical processes that comprise (cryptic) environmental sulfur cycling.

## Introduction

Cryptic sulfur cycling (i.e., the simultaneous reduction of sulfate and reoxidation of sulfide) has recently been observed in a variety of different environments from pelagic oxygen minimum zones (Canfield et al., 2010) to marine sediments (Holmkvist et al., 2011; Glombitza et al., 2016) and salt marshes (Mills et al., 2016). Moreover, experimental work in low sulfate, iron rich sediments indicates that an active sulfur cycle exists even in environments in which microbial iron reduction is expected to be favorable to sulfate reduction (Hansel et al., 2015). In all cases, this cycle involves the concomitant reduction of sulfate, via microbial sulfate reduction (MSR), and oxidation of the sulfide thereby produced. The cryptic nature arises because although the possible processes and intermediates involved with oxidative sulfur cycling are well characterized from both microbial and geochemical studies, it is unknown which processes and intermediates prevail in a particular environment. Sulfide oxidation can occur biotically as well as abiotically and results in the formation of a wide variety of inorganic intermediate species (${\text{S}}_{\text{x}}^{2-}$, S^0^, S_2_${\text{O}}_{3}^{2-}$, S_4_${\text{O}}_{6}^{2-}$, and ${\text{SO}}_{3}^{2-}$). Recent analytical advances have made accurate quantification of these compounds possible, however the low concentrations typically observed likely do not correlate with their biogeochemical importance (Zopfi et al., 2004).

The sulfur species formed during sulfide oxidation are dependent upon the physical and geochemical characteristics of a particular system, including the concentration of chemical oxidants [O_2_, Fe(III), and Mn(III, IV) oxides] and microbial community composition. Experimental work has indicated that under low oxidant to sulfide ratios, chemical sulfide oxidation by O_2_, FeOOH, and MnO_2_ typically yields zero-valent, or elemental, sulfur (ZVS, S^0^) as the primary oxidation product (Chen and Morris, 1972; O'Brien and Birkner, 1977; dos Santos Afonso and Stumm, 1992; Yao and Millero, 1996), due to thermodynamic and kinetic constraints on sulfide oxidation (Luther et al., 2011) and the stability of solid inorganic sulfur (orthorhombic S_8_) relative to other intermediates. Elemental sulfur is additionally formed during oxidation of sedimentary iron monosulfides (Pyzik and Sommer, 1981), through acid decomposition of polysulfides or thiosulfate (Dinegar et al., 1951; Chen and Gupta, 1973) and may also form during biological sulfide oxidation. Phototrophic sulfide oxidizing bacteria oxidize sulfide anaerobically in two steps, the first of which yields zero-valent sulfur (Frigaard and Dahl, 2008; Eddie and Hanson, 2013; Findlay et al., 2014). When sulfide is exhausted, these bacteria then oxidize ZVS to sulfate. Under O_2_ limiting conditions, ZVS may be formed during chemotrophic sulfide oxidation by O_2_ (Fisher et al., 1988; van den Ende and van Gemerden, 1993; Fuseler et al., 1996; Childress and Girguis, 2011). Chemotrophic sulfide oxidation using nitrate has furthermore been demonstrated to produce elemental sulfur as an intermediate during the complete oxidation of sulfide to sulfate (Fuseler et al., 1996). As a metastable intermediate, elemental sulfur is a common and widespread component of many aqueous (Ma et al., 2006; Li et al., 2008; Zerkle et al., 2010; Kamyshny et al., 2011; Findlay et al., 2014), sedimentary (Henneke et al., 1997; Zopfi et al., 2004; Yücel et al., 2010) and hydrothermal systems (Breier et al., 2012; Findlay et al., 2014). The speciation of this sulfur is likely heterogeneous: solid, orthorhombic S_8_ is the most stable form of ZVS; however, S^0^ produced by bacteria is typically more soluble and the speciation can vary among bacteria (Kleinjan et al., 2003). Moreover, nanoparticulate elemental sulfur (≤0.2 μm) has recently been observed to be a common component of a variety of sulfidic systems (Findlay et al., 2014).

Elemental sulfur formed either abiotically or microbially reacts with sulfide to form polysulfides (${\text{S}}_{\text{x}}^{2-}$), which are the most reduced of the intermediate species (Equation 1; Schwarzenbach and Fischer, 1960; Chen and Morris, 1972; Kleinjan et al., 2005). They consist of a chain of zero-valent sulfur atoms bound to a sulfur atom with the oxidation state (-II).

(1) $$x{\text{S}}^{0}+{\text{HS}}^{-}⇆{\text{S}}_{\text{x}}^{2-}$$

The reaction depicted in Equation (1) is a dynamic equilibrium in which ${\text{S}}_{\text{x}}^{2-}$ represents a spread of polysulfide species with different chain lengths (*x* is typically 2–9 in natural systems; Gun et al., 2004) in equilibrium with each other. Under equilibrium conditions the distribution of chain lengths can be predicted based upon chemical parameters (pH and the concentration of ZVS and sulfide; Kamyshny et al., 2004, 2007); however, non-equilibrium concentrations are frequently observed in natural systems (Kamyshny and Ferdelman, 2010; Lichtschlag et al., 2012). Polysulfides may also be formed directly as an enzymatic product of microbial metabolism (Griesbeck et al., 2000; c.f. Dahl, 2008; c.f. Findlay, 2016).

When oxidant concentrations (O_2_, MnO_2_) increase relative to sulfide, the predominant products of chemical sulfide oxidation appear to switch from elemental sulfur to thiosulfate and sulfite, with sulfate as the stable end product (Chen and Morris, 1972). Thiosulfate in particular has been shown to be a major oxidation product of sulfide in both freshwater and marine sediments (Jørgensen, 1990a,b). It is also the primary sulfur oxidation product of pyrite oxidation with Fe(III) (Luther, 1987) and can accumulate to high concentrations (≤ 100 μM) under intensive pyrite oxidation (Luther et al., 1986). MnO_2_ is also capable of oxidizing sulfide to thiosulfate and sulfate, likely through polysulfides as an intermediate (Burdige and Nealson, 1986). Thiosulfate is also produced biologically; for example during chemotrophic sulfide oxidation with oxygen (Childress et al., 1991; van den Ende and van Gemerden, 1993; Beinart et al., 2015) or during incomplete microbial sulfate reduction under substrate-limiting conditions (Vainshtein et al., 1980).

Thiosulfate is used widely in microbial sulfur metabolism by nearly all chemotrophic sulfur bacteria and many phototrophic bacteria (Alam et al., 2013). It has low chemical reactivity to oxygen, but may be oxidized by Fe(III) or MnO_2_ (Schippers and Jørgensen, 2002) to form tetrathionate, which may also form during pyrite oxidation (Luther et al., 1986). Tetrathionate is furthermore a common product of chemoheterotrophic microbial thiosulfate oxidation via thiosulfate dehydrogenase (Equation 2; Mason and Kelly, 1988)

(2) $$2\;{\text{S}}_{2}{\text{O}}_{3}^{2-}+2\;{\text{H}}_{2}\text{O}+{\text{O}}_{2}\to {\text{S}}_{4}{\text{O}}_{6}^{2-}+4\;{\text{OH}}^{-}$$

and of heterotrophic thiosulfate oxidation by denitrifying bacteria (Sorokin et al., 1999). Although it is observed in microbial culture (Tuttle and Jannasch, 1972; Mason and Kelly, 1988; Bak et al., 1993; van den Ende and van Gemerden, 1993), tetrathionate has only very rarely been detected in environmental samples (Podgorsek and Imhoff, 1999).

The most oxidized intermediate, sulfite, forms during sulfide oxidation at high oxidant to sulfide ratios (Chen and Morris, 1972; Zhang and Millero, 1993), but oxidizes quickly further to form ${\text{SO}}_{4}^{2-}$ (Zhang and Millero, 1991). Sulfite also reacts readily with organic matter (Vairavamurthy et al., 1994), and is oxidized, reduced and disproportionated by a variety of microorganisms for energy conservation (Janssen et al., 1996; Cypionka et al., 1998; Lie et al., 1999). Due to its high chemical and biological reactivity, it does not tend to accumulate to high concentrations in natural systems.

Once formed, each of these intermediate species may be microbially reduced, oxidized or disproportionated, or react further chemically. The prevalence of oxidative sulfur cycling indicated by observations of cryptic sulfur cycling and intermediate production combined with the diversity of microbial and geochemical processes involving these species clearly indicates the biogeochemical importance of these reactive intermediates; however, consumption rates in the environment are not well constrained. Rates are available for S_2_${\text{O}}_{3}^{2-}$ in freshwater and marine sediments (Jørgensen, 1990a,b; Zopfi et al., 2004) and for S_4_${\text{O}}_{6}^{2-}$ and ${\text{SO}}_{3}^{2-}$ in marine sediments (Podgorsek and Imhoff, 1999; Zopfi et al., 2004), but turnover rates for these species in aqueous environments and for ${\text{S}}_{\text{x}}^{2-}$ and S^0^ generally are lacking. The goal of the study presented here is therefore to constrain the rates of and processes broadly responsible for the turnover of all inorganic intermediate sulfur species (${\text{S}}_{\text{x}}^{2-}$, S^0^, S_2_${\text{O}}_{3}^{2-}$, S_4_${\text{O}}_{6}^{2-}$, ${\text{SO}}_{3}^{2-}$) in the sediments and water column of a freshwater lake. In order to minimize the effect of competing processes on rate measurements we use a series of incubation experiments in which each species is separately amended.

## Methodology

### Field setting and sampling

Lake Kinneret is a seasonally stratified freshwater lake located in northern Israel (32° 50′ N, 35° 35′E). The lake covers an area of 170 km^2^ and is 40 m deep at its deepest point (Station A). Thermal stratification begins around April and ends during the winter (December-January). During stratification, oxygen depletion and sulfate reduction in the water column and sediments lead to the prevalence of euxinic conditions and an active reoxidative sulfur cycle (Knossow et al., 2015).

Anoxic water samples for aqueous incubations were taken in May 2016 from the water column at Station A below the chemocline (defined as the point at which O_2_ became non-detectable, 17 m depth) at a water depth of 20 m. Oxygen concentrations were below detection (≤1 μM) and the sulfide concentration was 15 μM. Water was pumped from depth into covered glass containers that were sealed with a glass stopper, which prevented contamination of the samples by oxygen during transport to the laboratory, where the samples were processed the same day. Sediment samples for slurry incubations were taken from Station A in November 2016 from sediments underlying anoxic and sulfidic water. The cores were sealed, transported back to the laboratory, and processed the same day.

### Preparation of amendment solutions

H_2_S amendments were made from a stock solution of Na_2_S•9H_2_O, which was prepared in deoxygenated, deionised 18 MΩ (MilliQ®) water less than 1 h prior to addition. ${\text{SO}}_{3}^{2-}$, S_2_${\text{O}}_{3}^{2-}$, and S_4_${\text{O}}_{6}^{2-}$ amendments were also made from stock solutions of their sodium salts which were prepared in anoxic 18 MΩ water directly preceding addition to the experiment.

A polysulfide stock saturated with respect to elemental sulfur was made by preparing a mixture of 600 mM Na_2_S•9H_2_O and 6 M S^0^ in 50 mL deoxygenated deionized water. This solution was sealed, stirred and gently heated (40°C) for 4 h to (partially) dissolve elemental sulfur, then was allowed to stand overnight at room temperature to equilibrate. The pH was then adjusted to 7.4 (0.1–0.2 pH units lower than the pH of the experiments in order to prevent precipitation of S_8_ upon addition) and stood for another 2 h to allow S_8_ to precipitate and settle. The pH was confirmed, then the solution was filtered (0.2 μm) and used in experiments the same day.

Elemental sulfur colloids were prepared according to the method of Janek (1933) by the reaction of H_2_S with ${\text{SO}}_{3}^{2-}$ under acidic conditions. 3.6 g Na_2_SO_3_ and 6.5 g Na_2_S•9H_2_O were dissolved separately in 50 mL deionized water. 1.5 mL of the Na_2_SO_3_ solution was added to the sulfide solution, followed by about 8 mL H_2_SO_4_ (25 %) added dropwise until the cloudiness imparted to the solution upon addition of the acid barely disappeared after stirring. At this point, 3 mL concentrated H_2_SO_4_ were added to the Na_2_SO_3_ solution, which was then poured into the sulfide solution, turning the solution a milky yellow color. This mixture stood for 1 h, then was filtered through a Whatman (Size 5) 12.5 cm paper filter. The filtrate was washed with deionized water to remove soluble polythionates, then was resuspended in 300 mL deoxygenated deionized water for use in experiments. The colloids created by this synthesis were previously characterized by Steudel et al. (1988), who suggested a micellular structure and determined a formula of *x*(NaHSO_4_/Na_2_SO_4_)•*y*S_n_•*z*Na_2_S_m_O_6_ (*n* = 6–10, *m* = 4–16).

### Incubation experiments

Sediment slurry experiments were prepared using anoxic, non-sulfidic sediment at a dilution of 1:1 (v/v) with anoxic, sterile water taken from the overlying water column. The added water was heat sterilized (autoclaved to 120°C) before addition in order to isolate the effects of the sediment microbial community from those in the water column. The sediment slurries (pH 7.5) were prepared in a glovebag under an anoxic atmosphere and allowed to rest for at least 12 h prior to the injection of the amendment solutions. Injections of anoxic amendment solutions were made following the conditions outlined in Table 1. All experiments were conducted in duplicate. Following injection, the slurry experiments were incubated at 25°C, the normal temperature of the chemocline of Lake Kinneret in the summer (Knossow et al., 2015), in the dark under gentle shaking and were returned to the glovebag for sub-sampling.

**Table 1:** List of experimental conditions and species measured for each incubation.

	**Experiment Name**	**Conditions**	**Amendment**	**Sulfur species measured**
Aqueous	1A	Live	H_2_S, light	H_2_S, ${\text{S}}_{\text{x}}^{2-}$, ZVS, S_2_${\text{O}}_{3}^{2-}$, ${\text{SO}}_{3}^{2-}$
	1B	Killed		
	2A	Live	H_2_S, dark	H_2_S, ${\text{S}}_{\text{x}}^{2-}$, ZVS, S_2_${\text{O}}_{3}^{2-}$, ${\text{SO}}_{3}^{2-}$
	2B	Killed		
	3A	Live	${\text{S}}_{\text{x}}^{2-}$	H_2_S, ${\text{S}}_{\text{x}}^{2-}$, ZVS, S_2_${\text{O}}_{3}^{2-}$, ${\text{SO}}_{3}^{2-}$
	3B	Killed		
	4A	Live	S^0^ (colloidal)	H_2_S, ${\text{S}}_{\text{x}}^{2-}$, ZVS, S_2_${\text{O}}_{3}^{2-}$, ${\text{SO}}_{3}^{2-}$
	4B	Killed		
	5A	Live	S_2_${\text{O}}_{3}^{2-}$	H_2_S, S_2_${\text{O}}_{3}^{2-}$, ${\text{SO}}_{3}^{2-}$
	5B	Killed		
	6A	Live	S_4_${\text{O}}_{6}^{2-}$	H_2_S, S_2_${\text{O}}_{3}^{2-}$, ${\text{SO}}_{3}^{2-}$
	6B	Killed		
	7A	Live	${\text{SO}}_{3}^{2-}$	H_2_S, S_2_${\text{O}}_{3}^{2-}$, ${\text{SO}}_{3}^{2-}$
	7B	Killed		
Sediment	8	Live	H_2_S	H_2_S
	9	Live	${\text{S}}_{\text{x}}^{2-}$	H_2_S, ${\text{S}}_{\text{x}}^{2-}$, S_2_${\text{O}}_{3}^{2-}$, ${\text{SO}}_{3}^{2-}$
	10	Live	S^0^ (colloidal)	H_2_S, ZVS, S_2_${\text{O}}_{3}^{2-}$, ${\text{SO}}_{3}^{2-}$
	11	Live	S_2_${\text{O}}_{3}^{2-}$	H_2_S, S_2_${\text{O}}_{3}^{2-}$, S_4_${\text{O}}_{6}^{2-}$, ${\text{SO}}_{3}^{2-}$
	12	Live	S_4_${\text{O}}_{6}^{2-}$	H_2_S, S_2_${\text{O}}_{3}^{2-}$, S_4_${\text{O}}_{6}^{2-}$, ${\text{SO}}_{3}^{2-}$
	13	Live	${\text{SO}}_{3}^{2-}$	H_2_S, S_2_${\text{O}}_{3}^{2-}$, ${\text{SO}}_{3}^{2-}$
	14	Live	None	${\text{SO}}_{4}^{2-}$

Aqueous incubation experiments were set up in 150 mL glass vials sealed with rubber stoppers and aluminum caps. One hundred twenty-five milliliter of sample water (pH = 7.6) was transferred to each vial under an anoxic atmosphere in a glove bag and allowed to rest at least 12 h. We note that although the water contained 15 μM sulfide at the time of sampling, at the time the experiments were begun, the sulfide concentrations were between 0 and 5 μM, which are typical of interface environments. Experiments were begun upon syringe injection of the amended species through the rubber stopper. Non-sterilized experiments were conducted in triplicates and an abiotic control for each experiment was constructed by heat sterilization of the water under anoxic conditions (autoclaved to 120°C) prior to amendment. The initial concentration of each amended species was chosen to be as close to an environmentally relevant concentration range as possible, but high enough to allow accurate measurement of its consumption over time (Table 2). Throughout the experiments, oxygen was monitored in sub-samples using a fiberoptic optode (detection limit 1 μM; Firesting Pyroscience) and no oxygen was detected during either the preparation or course of the experiments. All experiments were stored in the dark unless otherwise noted (i.e., Experiment 1).

**Table 2:** Initial consumption rates and pseudo-first order rate constants for intermediate sulfur species from this work and the literature.

	**Species**	**C_0_ (μM)**	**Initial rate live (μM/hr)**	**Initial rate control (μM/h)**	**k′non-sterilized (h^−1^)**	**1σk′non-sterilized (h^−1^)**	**k′control (h^−1^)**	**Environment**	**References**
Water column	H_2_S (light)	300	6.9	1.3	0.0229	0.0163	0.0041	Freshwater water samples (Lake Kinneret)	This work
	H_2_S (dark)	300	0.58	0.42	0.0026	0.0011	0.0014		
	${\text{S}}_{\text{x}}^{2-}$-S		39	21	0.0103	0.0055	0.0081		
	S^0^	25	0.61	0.042	0.0527	0.0086	0.0016		
	S_2_${\text{O}}_{3}^{2-}$	125	3.0	0.29	0.023	0.0023	0.0017		
	S_4_ ${\text{O}}_{6}^{2-}$	120	0.5	0.031	0.0026	0.0003	0.0003		
	${\text{SO}}_{3}^{2-}$	150	48	8.0	0.0517	0.0027	0.0201		
Sediment	H_2_S	500	1,200		12.5	n/a		Freshwater sediment slurries (Lake Kinneret)	This work
	${\text{S}}_{\text{x}}^{2-}$		3,500		0.28	0.078			
	S^0^	100	26		0.86	0.04			
	S_2_${\text{O}}_{3}^{2-}$	140	1.2		0.0076	0.003			
	S_4_${\text{O}}_{6}^{2-}$	110	14		0.18	0.01			
	${\text{SO}}_{3}^{2-}$	130	22		0.2	0.02			
	${\text{SO}}_{4}^{2-}$	218	1.0		0.015	n/a			
	S_2_${\text{O}}_{3}^{2-}$	82	42		0.512			Black Sea sediment	Zopfi et al., 2004
		21	8.5		0.405			Black Sea sediment	Zopfi et al., 2004
		6	1.1		0.183			Black Sea sediment	Zopfi et al., 2004
		100	25		0.250			Odder River sediment	Jørgensen, 1990a
		125	69		0.552			Braband Lake sediment	Jørgensen, 1990a
		2	2.6		1.3			Hiddensee sediment	Podgorsek and Imhoff, 1999
	S_4_${\text{O}}_{6}^{2-}$	180	31.8		0.177			Weser Estuary (reduced)	Zopfi et al., 2004
		180	7.95		0.044			Weser Estuary (oxidized)	Zopfi et al., 2004
	${\text{SO}}_{3}^{2-}$	1.4	0.22		0.157			Black Sea sediment	Zopfi et al., 2004

*The rates for polysulfides from this work are expressed as total polysulfide*.

Throughout the course of all incubation experiments, sub-samples were taken and sulfur speciation was quantified for each experiment (Table 1). All sub-samples were taken in a glovebag under an anoxic atmosphere (<0.1% O_2_) to reduce the risk for oxygen contamination of the experiments and oxidation artifacts in the sub-samples. Sub-samples from the sediment slurries were taken using syringes and were filtered through a 0.45 μm prefabricated filter (Millipore) prior to analysis.

Due to the relatively long time scale of most experiments (days to week), it is probable that the microbial community changed in response to the substrate additions, and thus is not representative of *in situ* conditions. However, the lack of a lag phase in all incubations (with the exception of Experiment 6A) indicates that the capability to utilize these substrates is present and active, or is readily activated when they are provided.

### Analytical methods for sulfur speciation

Sulfide [operationally defined as S(-II) measured spectrophotometrically: ΣS(-II) = H_2_S + HS^−^ + polysulfide S(-II)] was preserved in zinc acetate (20 % w/v) and measured using the spectrophotometric method of Cline (1969) with detection at 665 nm. The method detection limit is 1 μM.

Polysulfides were quantified via HPLC following derivatisation with methyl triflate (Kamyshny et al., 2004, 2006). Briefly, 0.1 mL filtered (0.2 μM) sample, 0.1 mL phosphate buffer (pH 7.6), and 6 μL methyl triflate were added simultaneously to 0.8 mL methanol. The derivatised samples were stored at −20°C until analysis. Concentrations of polysulfides of chain lengths 2–8 were determined in derivatised samples by reversed phase HPLC with UV-detection at 220 and 230 nm. The method detection limit is 3–10 μM depending upon chain length (Kamyshny et al., 2004).

Zero-valent sulfur (colloidal and dissolved S^0^, polysulfide S^0^, S_4_${\text{O}}_{6}^{2-}$) was quantified as SCN^−^ by HPLC using a C-30 column modified with polyethylene glycol (5%) (Rong et al., 2005; Kamyshny, 2009). ZVS was converted to SCN^−^ via cyanolysis by injecting 5–10 mL of sample and 20 μL KCN (10% w/v) concurrently into 20 mL of boiling boric acid (1% w/v), after which the solution was returned to a boil and the volume was reduced to 5–10 mL. Corrections were made for contributions from S_4_${\text{O}}_{6}^{2-}$ and ${\text{S}}_{\text{x}}^{2-}$, therefore ZVS concentrations presented here represent only dissolved and colloidal S^0^. Cyanolysis was chosen over extraction by organic solvents (e.g., chloroform or toluene) as determination of cyanide-reactive sulfur yields higher recovery of ZVS in Lake Kinneret waters, perhaps due to the presence of biologically produced hydrophilic sulfur (Knossow et al., 2015).

Tetrathionate was quantified immediately after sub-sampling in filtered samples using the same HPLC method described above for cyanide-reactive ZVS samples. The detection limit for this method is 0.5 μM. Tetrathionate concentrations in filtered subsamples were found to be stable over time scales of 1–3 h, which allowed sufficient time for accurate measurement by HPLC.

Thiosulfate and sulfite were quantified by HPLC following derivatisation by monobromobimane (Newton et al., 1981; Zopfi et al., 2004). The method detection limit for both S_2_${\text{O}}_{3}^{2-}$ and ${\text{SO}}_{3}^{2-}$ is 0.005 μM.

Sulfate concentrations were measured by ion chromatography (Metrohm) with a conductivity detector after filtration and dilution of sub-samples preserved in zinc acetate. Sodium carbonate/bicarbonate buffer was used as the eluent. The detection limit for this method is 10 μM.

### Rate calculations

All reactions were treated as pseudo-first order and consumption rates were calculated based upon the method of initial rates. Pseudo-first order behavior and kinetic constants were derived from plots of ln(concentration) over time.

This treatment of the data means that the kinetics may be described after the rate law given by Equation (3):

(3) $$\frac{dC}{dt}=k^{\prime }[C]$$

where C is the concentration of a particular species and *k*′ is the pseudo-first order rate constant. Equation 3 can be integrated to give Equation (4).

(4) $$C_{t}=C_{0}e^{-kt}$$

This assumes that the kinetics are dependent upon the concentration of the species of interest, and that all other factors are constant during the experiment (e.g., microbial biomass, chemical oxidants). As the electron donor or acceptor is unknown for the consumption reactions observed in these experiments, this yields the most realistic representation of the decomposition kinetics. It is important to consider, however, that as most of the reactions examined here are microbially mediated, it is therefore likely that the actual kinetics have a relationship to the substrate concentration more consistent with a Michaelis-Menten description.

## Results

### Sediment experiments

#### Sulfide, polysulfide, and ${\text{S}}_{\text{colloidal}}^{0}$

**Figure 1:**
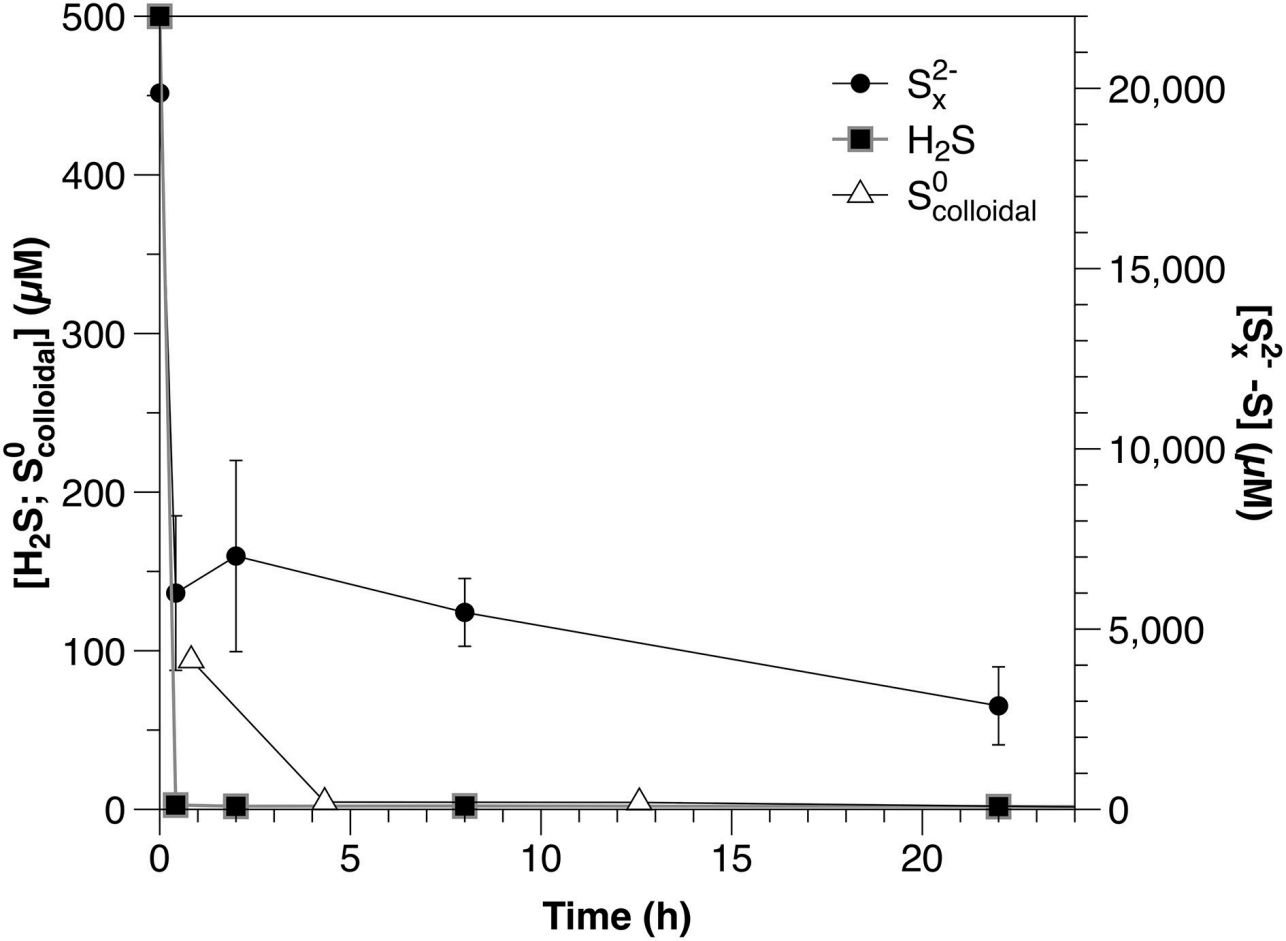
Timecourse for sulfide, polysulfide and ${\text{S}}_{\text{colloidal}}^{0}$ from separately amended sediment slurry incubation experiments.

Concentrations of all reduced sulfur species (H_2_S, ${\text{S}}_{\text{x}}^{2-}$, ${\text{S}}_{\text{colloidal}}^{0}$) decreased rapidly within the first hour after amendment (Figure 1; Table 2). Both sulfide and ${\text{S}}_{\text{colloidal}}^{0}$ were depleted to below the detection limits of the relevant analytical methods; however, after initial rapid consumption, polysulfide concentrations continued to decrease slowly during the remainder of the experiment.

#### Thiosulfate

**Figure 2:**
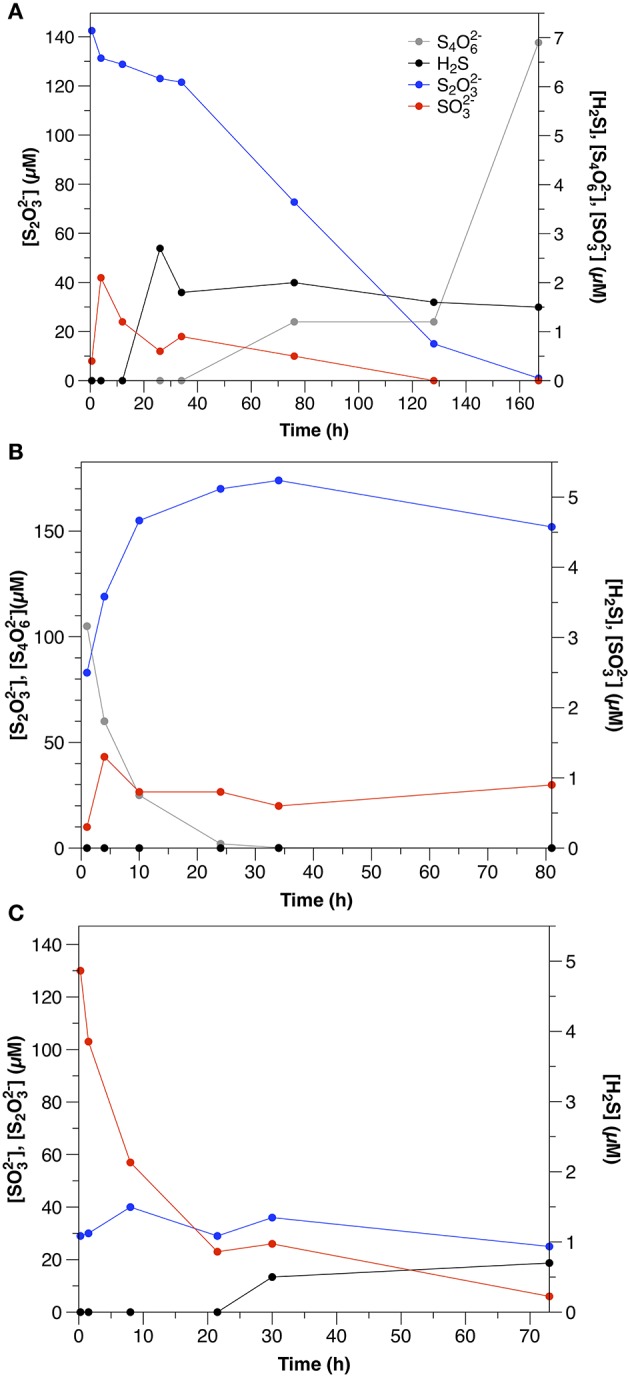
Sulfur speciation in slurry experiments amended with **(A)** thiosulfate (Exp. 11), **(B)** tetrathionate (Exp. 12), and **(C)** sulfite (Exp. 13).

S_2_${\text{O}}_{3}^{2-}$ was consumed at an initial rate of 1.2 μM h^−1^. The initial consumption of S_2_${\text{O}}_{3}^{2-}$ was accompanied by a small increase in ${\text{SO}}_{3}^{2-}$ concentrations, which then decreased throughout the remainder of the experiment (Figure 2A). After the initial 12 h, low concentrations (1–2 μM) of sulfide were also observed. S_4_${\text{O}}_{6}^{2-}$ also appeared after 34 h, with concentrations increasing to nearly 7 μM by the end of the experiment.

#### Tetrathionate

S_4_${\text{O}}_{6}^{2-}$ disappeared at a rate of 14.3 μM h^−1^, which was accompanied by a concomitant increase in t S_2_${\text{O}}_{3}^{2-}$ (Figure 2B). ${\text{SO}}_{3}^{2-}$ concentrations were low throughout the course of the experiments (< 1.5 μM), and sulfide was not detected.

#### Sulfite

${\text{SO}}_{3}^{2-}$ was consumed at an initial rate of 21.7 μM h^−1^ (Figure 2C). After the first day, low concentrations of sulfide were observed (<1.5 μM). In contrast to water column experiments (Section Thiosulfate), S_2_${\text{O}}_{3}^{2-}$ concentrations were constant at about 30 μM throughout the course of the experiment.

#### Sulfate reduction

A control experiment (i.e., no amendment) was set up in which ${\text{SO}}_{4}^{2-}$ concentrations were measured over time in order to estimate the sulfate reduction rate. The initial ${\text{SO}}_{4}^{2-}$ concentration in the experiments was 218 μM, which decreased to 143 μM over 3 days, yielding an apparent sulfate reduction rate of 1.02 μM h^−1^. No precipitation of sulfate is expected at the concentrations present in the experiments.

### Water column experiments

#### Sulfide and polysulfide

**Figure 3:**
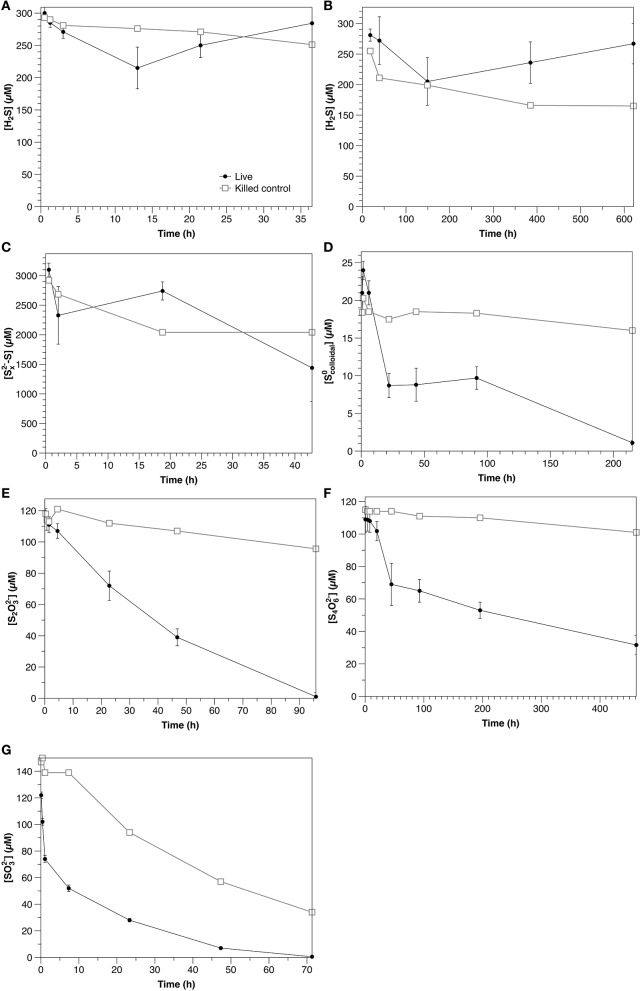
Time course of water column amendment experiments in live and killed control experiments. **(A)** Exp. 1; **(B)** Exp. 2; **(C)** Exp. 3; **(D)** Exp. 4; **(E)** Exp. 5; **(F)** Exp. 6; **(G)** Exp. 7. Error bars represent three independent replicates. Please note that the scales of both the concentration (y) and time (x) axes change for each experiment.

Under ambient laboratory light conditions, sulfide decreased with an initial rate of 6.9 μM hr^−1^ in non-sterilized experiments and 1.3 μM h^−1^ in the control (Figure 3A). In dark experiments, sulfide loss rates were 0.58 μM h^−1^, with no significant difference between non-sterilized and sterilized experiments (Figure 3B). After 200 h, however, sulfide concentrations were observed to increase in non-sterilized experiments, likely due to microbial sulfate reduction.

Polysulfide concentrations (presented as the sum of sulfur atoms present in polysulfides rather than of individual chains) decreased at an initial rate of 39 μM h^−1^ in non-sterilized experiments, with no significant difference with respect to the control (Figure 3C).

#### Zero-valent sulfur (${\text{S}}_{\text{colloidal}}^{0}$)

Colloidal zero-valent sulfur in non-sterilized experiments decreased at an initial rate of 0.61 μM h^−1^ with no lag time. In controls, sulfur concentrations were constant over the course of the experiment (within the 15% error of the measurement; Kamyshny, 2009), indicating the absence of abiotic oxidation or coagulation (Figure 3D).

**Figure 4:**
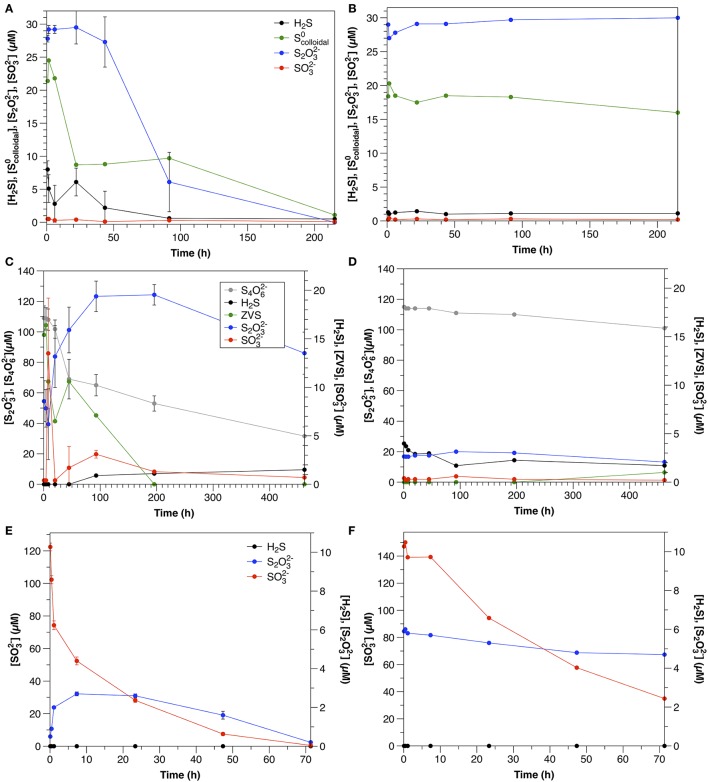
Sulfur dynamics in experiments amended with colloidal sulfur (A = Exp 4A; B = Exp 4B), tetrathionate (A = Exp 6A; B = Exp 6B) and sulfite (A = Exp 7A; B = Exp 7B). Sulfide concentrations in Experiment 7 were below detection (1 μM) at all times. Error bars represent three independent replicates.

S_2_${\text{O}}_{3}^{2-}$ was initially present in the colloidal sulfur amendments to both non-sterilized and control experiments (Figures 4A,B), likely remaining from the synthesis (Steudel et al., 1988), despite washing during the preparation. In non-sterilized experiments, S_2_${\text{O}}_{3}^{2-}$ decreased after a lag time of about 22 h at a rate of 0.2 μM h^−1^. S_2_${\text{O}}_{3}^{2-}$ concentrations in the control did not change over the course of the experiment.

In non-sterilized experiments, sulfide was present initially (8 μM), likely due to ongoing sulfate reduction, but decreased throughout the course of the experiment. In the control experiment, sulfide was present at about 1 μM and concentrations did not change significantly throughout the course of the experiment. In both the non-sterilized experiments and controls ${\text{SO}}_{3}^{2-}$ concentrations were low and stable over the course of the experiment.

#### Thiosulfate

S_2_${\text{O}}_{3}^{2-}$ was consumed in non-sterilized experiments with no lag time at an initial rate of 3.0 μM h^−1^. In the control, S_2_${\text{O}}_{3}^{2-}$ concentrations declined slowly over the course of the experiment at a rate of 0.29 μM hr^−1^ (Figure 3E). No oxidation products were detected in non-sterilized S_2_${\text{O}}_{3}^{2-}$-amended incubations. A slight increase in ${\text{SO}}_{3}^{2-}$ (to 2.3 μM) was observed in the control, likely reflecting a slow oxidation of S_2_${\text{O}}_{3}^{2-}$ (*data not shown*).

#### Tetrathionate

In non-sterilized experiments, tetrathionate consumption began after a lag period (≤20 h). After this point, concentrations decreased with an initial rate of 0.50 μM h^−1^. In contrast, in the control, tetrathionate was relatively stable and declined at a rate of 0.031 μM h^−1^ (Figure 3F). In non-sterilized experiments, S_2_${\text{O}}_{3}^{2-}$ increased with initial tetrathionate consumption, then slowly decreased (Figure 4B), whereas the concentrations of all measured sulfur species were stable in the control (Figure 4D).

#### Sulfite

${\text{SO}}_{3}^{2-}$ was consumed with no lag time in both non-sterilized experiments (48 μM h^−1^) and controls (8.0 μM h^−1^) (Figure 3G). In both non-sterilized and control experiments, most ${\text{SO}}_{3}^{2-}$ was not recovered in the measured sulfur pools (sulfide or S_2_${\text{O}}_{3}^{2-}$). Low concentrations of S_2_${\text{O}}_{3}^{2-}$ were measured at the initial time point in both sterilized and non-sterilized experiments, which may be due to impurities in the ${\text{SO}}_{3}^{2-}$ solution, or reaction of ${\text{HSO}}_{3}^{-}$ (pK_a_ = 6.97) with low amounts of sulfide upon amendment (Heunisch, 1977). Formation of additional S_2_${\text{O}}_{3}^{2-}$ during the time course was observed only in non-sterilized experiments (Figure 4E).

#### Sulfate reduction

An increase in sulfide concentration attributed to sulfate reduction was observed in Experiment 2A after 200 h. Assuming that sulfide oxidation continued at the same initial rate, an apparent sulfate reduction rate of 0.8 μM h^−1^ may be estimated.

## Discussion

### Turnover of sulfide and polysulfide

During sediment incubations, the extremely rapid initial consumption of sulfide and polysulfide was likely due to abiotic reaction with iron minerals. The reactive iron content ranges from 50 to 100 μM Fe g^−1^ (dry weight) at Station A (Eckert, 2000) and the formation of iron-sulfides is further suggested by the change in the sediment color from brown to gray/black during these incubations. The slower decrease in polysulfide concentrations after the initial consumption, however, may be due to either continued reaction with less reactive iron minerals or microbial consumption. Further work is required to constrain this, as polysulfides play an integral role in pyrite formation (Rickard and Luther, 2007), isotope exchange processes between reduced sulfur species (Fossing and Jørgensen, 1990; Kamyshny et al., 2014), and furthermore represent an important potential energy source for micro-organisms (Findlay, 2016).

It is important to note that the results of the sulfide and polysulfide incubation experiments are not directly comparable. Polysulfide concentrations are dependent upon sulfide and the rapid scavenging of sulfide by these sediments would result in a corresponding decrease in polysulfides. Therefore, the consumption rate of polysulfides should equal to or higher than that of sulfide under similar conditions. To avoid this being the primary process affecting polysulfide dynamics in these experiments, however, polysulfides were added at concentrations much higher than those of sulfide (Figure 1). The result of this is that polysulfide consumption appears slower relative to sulfide when corrected for concentration (Table 2), but this is an artifact of the experimental conditions and not an accurate description of the relative reactivity.

In water column samples, a clear biological influence on sulfide oxidation was observed in the light (Figure 3A; Table 2) over the dark rate in Experiment 2A, indicating that sulfide was oxidized phototrophically (either through phototrophic sulfur bacteria, or cyanobacteria, both of which are typically present at the chemocline, e.g., Rimmer et al., 2008). Despite this clear biological influence, the production of S_2_${\text{O}}_{3}^{2-}$, the main oxidation product, was similar between the non-sterilized (0.2 μM h^−1^) and control (0.3 μM h^−1^) experiments (*data not shown*). This could be due to internal storage of oxidation products by phototrophic sulfur bacteria (van Gemerden, 1986), so that the solution chemistry reflects only the chemical oxidation in both instances. During sulfide oxidation under dark conditions in the sterilized control, S_2_${\text{O}}_{3}^{2-}$ concentrations increase with time in the control at a rate of 0.41 μM h^−1^, whereas in the non-sterilized experiment they decrease at a rate of 0.051 μM h^−1^ (*data not shown*), suggesting that although a biological impact on sulfide oxidation was not observed, microbial consumption of the products from the chemical oxidation occurred.

Polysulfide consumption in the aqueous experiments appears to be abiotic. Although the consumption rate in the non-sterilized experiments (39 μM h^−1^) was higher than that in the control (21 μM h^−1^), when variability between the triplicate non-sterilized experiments is accounted for, there is no statistical difference between the two rates (Table 2). The interpretation of these experiments is however complicated by the possibility of concurrent sulfate reduction (e.g., Figure 3B), which would strongly impact the concentration of sulfide and thus also polysulfide (Equation 1). If indeed there is biological oxidation, this could be partially masked by sulfate reduction in the non-sterilized experiments. Intriguingly, in the non-sterilized experiments S_2_${\text{O}}_{3}^{2-}$ concentrations consistently increased throughout the incubation period (from 6.7 ± 0.7 to 120 ± 60 μM), whereas in the control, concentrations were stable (2.3 ± 0.6 μM). This is in contrast to the sulfide oxidation experiments (Experiment 2), in which thiosulfate concentrations increased in the control and decreased in the non-sterilized experiments, and may therefore be a consequence of biological polysulfide oxidation, rather than sulfide oxidation.

### ${\text{S}}_{\text{colloidal}}^{0}$ consumption

This is the first report of directly measured rates of ${\text{S}}_{\text{colloidal}}^{0}$ consumption under environmentally relevant conditions, although previous studies have indicated pelagic (Jørgensen et al., 1979) and sedimentary (Fossing and Jørgensen, 1990) S^0^ consumption. In the sediment incubations, rapid ${\text{S}}_{\text{colloidal}}^{0}$ consumption similar to that observed for sulfide and polysulfide was likely abiotic, due to reaction with reactive iron minerals (pyritisation or absorption).

In the water column experiments, (Experiment 3A), ${\text{S}}_{\text{colloidal}}^{0}$ consumption was the fastest of all amended species (normalized for concentration) and was biologically mediated (Figure 4A). One interesting feature of these experiments is that S_2_${\text{O}}_{3}^{2-}$ was also present in this amendment as a by-product of the synthesis, however, the rate of S_2_${\text{O}}_{3}^{2-}$ consumption in Experiment 3A is slower than expected based upon the kinetics determined from Experiment 5A (0.69 μM h^−1^ ± 0.068). Moreover, a lag time was not observed in Experiment 5A (Figure 3E), as was observed in Experiment 3A. This, and a pause in ${\text{S}}_{\text{colloidal}}^{0}$ consumption once S_2_${\text{O}}_{3}^{2-}$ consumption began (Figure 4A) indicate that a similar mechanism was responsible for both the use of both ${\text{S}}_{\text{colloidal}}^{0}$ and S_2_${\text{O}}_{3}^{2-}$, and that consumption of both species could not occur simultaneously.

One possible explanation for this observation may be the speciation of the sulfur colloids themselves. At 22 h, when S_2_${\text{O}}_{3}^{2-}$ consumption began, approximately 41 ± 4% of the ${\text{S}}_{\text{colloidal}}^{0}$ had been consumed. This corresponds to the characterization of this synthesis by Steudel et al. (1988), which demonstrated that about 45% of the S^0^ present in the sols was in a form other than S_8_, likely longchain polythionates. We thus hypothesize that this more reactive S^0^ was consumed first, followed by a switch to thiosulfate metabolism, and then again back to the remaining, less reactive ZVS (present as S_8_) once thiosulfate concentrations were low. The second phase of ${\text{S}}_{\text{colloidal}}^{0}$ consumption occurred three times slower than the initial consumption, further suggesting that two different species were involved. In contrast, S_2_${\text{O}}_{3}^{2-}$ concentrations were stable over the course of the experiment in the control (Figure 4B).

### Connection between thiosulfate and tetrathionate cycling

Consumption of S_2_${\text{O}}_{3}^{2-}$ and S_4_${\text{O}}_{6}^{2-}$ in the water column was largely a biological process (>90%; Figures 3E,F, Table 2). Sedimentary consumption was also likely predominantly biological, as both of these species are chemically stable under anoxic, non-sulfidic conditions. Notably, S_4_${\text{O}}_{6}^{2-}$ consumption was slower than S_2_${\text{O}}_{3}^{2-}$ consumption in the water column, whereas the opposite was true for sediment incubations. The rates of S_2_${\text{O}}_{3}^{2-}$ consumption observed in this study are notably lower than previous reports from both freshwater and marine sediments (Jørgensen, 1990a,b; Elsgaard and Jørgensen, 1992; Zopfi et al., 2004).

No oxidation products were observed in non-sterilized S_2_${\text{O}}_{3}^{2-}$ amendments in aqueous experiments. This could indicate oxidation to either S_4_${\text{O}}_{6}^{2-}$ or ${\text{SO}}_{4}^{2-}$ (which were not measured in these experiments), consistent with microbial S_2_${\text{O}}_{3}^{2-}$ oxidation in chemoheterotrophic bacteria. Disproportionation could also have occurred, followed by oxidation of sulfide (as discussed in the following section for ${\text{SO}}_{3}^{2-}$ amendments). In sediment incubation experiments, however, measurements of S_4_${\text{O}}_{6}^{2-}$ were made, and production was observed after about 35 h of incubation, indicating that S_2_${\text{O}}_{3}^{2-}$ oxidation to S_4_${\text{O}}_{6}^{2-}$ was responsible for at least part of the observed S_2_${\text{O}}_{3}^{2-}$ consumption, similar to experiments conducted in coastal sediments by Podgorsek and Imhoff (1999), in which S_4_${\text{O}}_{6}^{2-}$ accounted for up to 57% of oxidized S_2_${\text{O}}_{3}^{2-}$. In our case, assuming that S_4_${\text{O}}_{6}^{2-}$ was only produced and not consumed, oxidation of S_2_${\text{O}}_{3}^{2-}$ to S_4_${\text{O}}_{6}^{2-}$ accounts for 10% of S_2_${\text{O}}_{3}^{2-}$ loss in the experiments. The remainder may be due to either reduction or disproportionation; previous studies using radio-labeled S_2_${\text{O}}_{3}^{2-}$ tracers demonstrated simultaneous oxidation, reduction and disproportionation throughout the sediment column (Fossing and Jørgensen, 1990).

This is the first report of S_4_${\text{O}}_{6}^{2-}$ consumption in aqueous environmental samples, and although the rate is slower than that observed in sediment incubations (this study, Zopfi et al., 2004), it is consistent with previous reports in its stoichiometry and microbial nature. S_4_${\text{O}}_{6}^{2-}$ can be reduced by a variety of different microorganisms, including but not limited to sulfate-reducing bacteria (c.f. Barrett and Clark, 1987). In the non-sterilized, aqueous incubations (Experiment 6A), S_4_${\text{O}}_{6}^{2-}$ consumption was accompanied by a nearly stoichiometric increase in S_2_${\text{O}}_{3}^{2-}$ (1.9; Figure 4C). This stoichiometry is consistent with S_4_${\text{O}}_{6}^{2-}$ reduction via tetrathionate reductase (Bak et al., 1993) after Equation (5).

(5) $${\text{S}}_{4}{\text{O}}_{6}^{2-}+2[\text{H}]\to 2\;{\text{S}}_{2}{\text{O}}_{3}^{2-}+2\;{\text{H}}^{+}$$

where [H] represents the enzyme tetrathionate reductase. Based upon S_2_${\text{O}}_{3}^{2-}$ production, Equation (5) can account for 100% of S_4_${\text{O}}_{6}^{2-}$ consumption in these experiments.

S_4_${\text{O}}_{6}^{2-}$ also reacts with sulfide, forming S^0^ and which would also result in a 2:1 S_2_${\text{O}}_{3}^{2-}$: S_4_${\text{O}}_{6}^{2-}$ stoichiometry (Equation 6; Sorokin et al., 1996; Podgorsek and Imhoff, 1999; Zopfi et al., 2004).

(6) $${\text{HS}}^{-}+{\text{S}}_{4}{\text{O}}_{6}^{2-}\to 2\;{\text{S}}_{2}{\text{O}}_{3}^{2-}+{\text{S}}^{0}+{\text{H}}^{+}$$

ZVS concentrations were highest at the start of the experiments (15 μM), before significant S_4_${\text{O}}_{6}^{2-}$ consumption was observed. In order to explain ZVS formation by Equation (6), 15 μM S_4_${\text{O}}_{6}^{2-}$ would need to have been consumed initially, which is not supported by the data. ZVS has been also found to form from the decomposition of S_2_${\text{O}}_{3}^{2-}$ due to acid generation by S_4_${\text{O}}_{6}^{2-}$ oxidation (Bak et al., 1993), however this is also not likely at the pH and buffering capacity of these experiments. This points to a source of ZVS not related to the microbial metabolism of S_4_${\text{O}}_{6}^{2-}$ (likely oxidation of sulfide in the initial sample prior to the commencement of the experiment) and indicates that S_4_${\text{O}}_{6}^{2-}$ consumption is due to microbial tetrathionate reduction via Equation (5).

Although S_2_${\text{O}}_{3}^{2-}$ was still the dominant product of tetrathionate consumption in the sediment slurry incubations (Figure 2B), the ratio between S_2_${\text{O}}_{3}^{2-}$ production and S_4_${\text{O}}_{6}^{2-}$ consumption was lower (0.5) than observed in the aqueous incubations. S_2_${\text{O}}_{3}^{2-}$ production after Equation (5) therefore accounts for only 25% of S_4_${\text{O}}_{6}^{2-}$ consumption. A lower ratio (1.5) results from S_4_${\text{O}}_{6}^{2-}$ disproportionation (Equation 7; Zopfi et al., 2004), however this process is also not consistent with the stoichiometry observed in the sediments.

(7) $$4\;{\text{S}}_{4}{\text{O}}_{6}^{2-}+4\;{\text{H}}_{2}\text{O}\to 6\;{\text{S}}_{2}{\text{O}}_{3}^{2-}+{\text{S}}_{3}{\text{O}}_{6}^{2-}+{\text{SO}}_{4}^{2-}+8\;{\text{H}}^{+}$$

There are several possible explanations for the lower production of S_2_${\text{O}}_{3}^{2-}$ than expected based upon the reaction stoichiometries in Equations (5, 7) that was observed in the sediment incubations. First, S_4_${\text{O}}_{6}^{2-}$ reacts with sulfide (Equation 6). Although sulfide was likely produced by microbial sulfate reduction, it was not observed in any of the sediment incubation experiments, as these sediments demonstrate very effective sulfide scavenging (Figure 1). Furthermore, apparent sulfate reduction rates were lower than S_4_${\text{O}}_{6}^{2-}$ consumption, similar to the observation made by Zopfi et al. (2004). It is therefore unlikely that Equation (6) was the dominant consumption pathway for S_4_${\text{O}}_{6}^{2-}$ in these experiments. It is more likely that S_2_${\text{O}}_{3}^{2-}$ formed during S_4_${\text{O}}_{6}^{2-}$ consumption was consumed biologically (e.g., via reduction or disproportionation).

Equations (2, 5) illustrate the interconnectivity between S_2_${\text{O}}_{3}^{2-}$ and S_4_${\text{O}}_{6}^{2-}$ production and consumption, as each is essentially the reverse of the corresponding process. Thermodynamic calculations indicate that consumption of S_4_${\text{O}}_{6}^{2-}$ is favorable to consumption of S_2_${\text{O}}_{3}^{2-}$ at all but very low ratios of S_4_${\text{O}}_{6}^{2-}$ to S_2_${\text{O}}_{3}^{2-}$ (≤1 × 10^−9^). Thus, in natural environments the relative concentrations of S_2_${\text{O}}_{3}^{2-}$ and S_4_${\text{O}}_{6}^{2-}$ should control the direction in which this reaction proceeds. It has been known from microbiological studies that the cycles of S_2_${\text{O}}_{3}^{2-}$ and S_4_${\text{O}}_{6}^{2-}$ are connected (Tuttle and Jannasch, 1972, 1973; Barrett and Clark, 1987; Bak et al., 1993), however, the role of S_4_${\text{O}}_{6}^{2-}$ in microbial metabolism and in the sedimentary sulfur cycle is still not clear. Nevertheless, the importance of S_2_${\text{O}}_{3}^{2-}$ metabolism has long been established in both freshwater (Jørgensen, 1990a) and marine (Jørgensen, 1990b) sediments. The observation of S_4_${\text{O}}_{6}^{2-}$ production during S_2_${\text{O}}_{3}^{2-}$ metabolism in this study and by Podgorsek and Imhoff (1999) thus points to a potential source of tetrathionate in sediments. Although the concentrations of S_2_${\text{O}}_{3}^{2-}$ used in these experiments are much higher than those found in most sedimentary environments (which likely is the cause for the production of detectable tetrathionate production), it is nevertheless likely that tetrathionate is produced under normal environmental conditions and Podgorsek and Imhoff (1999) observed its formation even in sulfidic sediments.

### Sulfite consumption

Based upon free energy yields, ${\text{SO}}_{3}^{2-}$ disproportionation (−233 kJ mol^−1^; Equation 8) is expected to be the favored metabolism under anoxic conditions, followed by reduction via sulfite reductase (−171 kJ mol^−1^; Equation 9, Krämer and Cypionka, 1989).

(8) $$4\;{\text{SO}}_{3}^{2-}+{\text{H}}^{+}\to 3\;{\text{SO}}_{4}^{2-}+{\text{HS}}^{-}$$

(9) $${\text{SO}}_{3}^{2-}+3{\text{H}}_{2}+{\text{H}}^{+}\to {\text{HS}}^{-}+3{\text{H}}_{2}\text{O}$$

Sulfide was detected in neither sediment incubations nor non-sterilized and control water column experiments; however, the presence of S_2_${\text{O}}_{3}^{2-}$ in both sediment (Exp 13, Figure 2C) and non-sterilized water column (Experiment 7, Figure 4C) experiments may indicate its formation, as ${\text{SO}}_{3}^{2-}$ reacts with sulfide to form S_2_${\text{O}}_{3}^{2-}$ via equation 10 with a free energy yield of −167 kJ/mol (Krämer and Cypionka, 1989).

(10) $$4\;{\text{HSO}}_{3}^{-}+2\;{\text{H}}_{2}\text{S}\to \;3\;{\text{S}}_{2}{\text{O}}_{3}^{2-}+3\;{\text{H}}_{2}\text{O}$$

For water column experiments, based upon the rates of sulfide oxidation in the dark determined from Experiment 2, we calculate that anaerobic sulfide oxidation could account for the loss of all potential sulfide formed via disproportionation, thus masking its formation. The absence of major electron acceptors (O_2_, Fe(III), MnO_2_, ${\text{NO}}_{3}^{-}$) in these experiments makes disproportionation the most likely process responsible for ${\text{SO}}_{3}^{2-}$ consumption within the water column. The capacity for ${\text{SO}}_{3}^{2-}$ disproportionation is found among sulfate-reducing bacteria, which are known to be present in the chemocline of the lake (Eckert and Conrad, 2007). In sediment experiments, the extremely rapid removal of sulfide observed in Experiment 8 (Figure 1) can explain the very low sulfide concentrations measured in Experiment 13, making sulfite loss consistent with either disproportionation or reduction in both cases. The capacity for dissimilatory sulfite reduction appears to be predominantly found in sulfate-reducing bacteria (Barrett and Clark, 1987).

An alternative possibility is ${\text{SO}}_{3}^{2-}$ oxidation by sulfite oxioreductase via Equation (11), however, this reaction yields a much lower energy (−19.7 kJ mol^−1^) than disproportionation (Krämer and Cypionka, 1989) and is thus a less likely explanation.

(11) $${\text{SO}}_{3}^{2-}+{\text{H}}_{2}\text{O}\to {\text{SO}}_{4}^{2-}+{\text{H}}_{2}$$

### Implications for sulfur cycling in natural systems

The results of these experiments provide several important insights into the cycling of intermediate sulfur species in natural systems and the biogeochemical controls on their consumption. First, consumption of all intermediate species, with the exception of polysulfide, appears to be predominantly biologically mediated in anoxic aqueous environments with low concentrations of trace metals (Fe ≤ 1 μM). Polysulfide concentrations will be controlled by their formation rate from sulfide and S^0^, balanced by geochemical and possibly biological consumption and are often not in equilibrium with S^0^ (Kamyshny and Ferdelman, 2010; Lichtschlag et al., 2012). As the water-columns of most stratified, non-polluted natural environments have low trace metal content, these results are widely applicable.

Second, in anoxic, non-sulfidic sediments, consumption of S_2_${\text{O}}_{3}^{2-}$ and S_4_${\text{O}}_{6}^{2-}$ will likely be microbially mediated, ${\text{SO}}_{3}^{2-}$ consumption will likely be both microbial and chemical (e.g., through reaction with organic matter) and the concentrations of S^0^ will be controlled by the presence and speciation of reactive iron (e.g., through pyrite formation). A comparison between the turnover rates measured here for S_2_${\text{O}}_{3}^{2-}$, S_4_${\text{O}}_{6}^{2-}$, and ${\text{SO}}_{3}^{2-}$ and those measured in previous studies from both freshwater and marine sediments (Table 2) shows that the rates measured for Lake Kinneret are generally lower, however the sequence of reactivity for these species, namely ${\text{SO}}_{3}^{2-}$ ≈ S_4_${\text{O}}_{6}^{2-}$ > S_2_${\text{O}}_{3}^{2-}$ is the same. Therefore, the patterns determined here likely also apply to the anoxic, non-sulfidic sediments that compose a large portion of coastal marine environments (Zopfi et al., 2004). In sulfidic sediments, S_4_${\text{O}}_{6}^{2-}$ and ${\text{SO}}_{3}^{2-}$ may react with sulfide, increasing the abiotic component of the consumption rate. In contrast, S_2_${\text{O}}_{3}^{2-}$ is produced even in sulfidic systems (Jørgensen, 1990b). Notably, the presence of sulfide will impact the thermodynamics of particular reactions, for example disproportionation of S^0^ or S_2_${\text{O}}_{3}^{2-}$ (Jørgensen and Bak, 1991).

Third, the speciation of zero-valent or elemental sulfur in a particular environment will expectedly have a strong impact on its reactivity. Therefore, bulk descriptions of S^0^ based upon extraction (e.g., in toluene or methanol) or even reactivity (cyanide-reactive sulfur) give only limited information regarding the biogeochemical reactivity or microbial availability of the measured sulfur in a system. The results of this study and previous experiments indicate that in sediments, elemental sulfur reacts quickly, either with iron to form pyrite (c.f. Rickard and Luther, 2007) or due to absorption onto mineral surfaces (Fossing et al., 1992). In contrast, in the water column, ${\text{S}}_{\text{colloidal}}^{0}$ consumption is biological, with no significant chemical reactivity observed. ${\text{S}}_{\text{colloidal}}^{0}$ is furthermore the most rapidly consumed species in aqueous experiments, indicating its potential importance as a microbial substrate. In contrast, S^0^ often accumulates in anoxic water columns and in marine sediments, suggesting that either its formation occurs rapidly, or that it is present in a less reactive form than the colloids synthesized here (e.g., as crystalline orthorhombic sulfur). This may also be impacted by how the S^0^ forms. Biologically produced S^0^ may be more hydrophilic and closer in reactivity to the colloids prepared here (Zöphel et al., 1988), and so would turn over rapidly, whereas inorganically produced S^0^ (e.g., from sulfide oxidation by oxide minerals) is hydrophobic, less reactive and therefore accumulates to larger crystals. This would indicate that it is the structure and bonding environment of the initial phases and not the size that control the reactivity.

Finally, we observe that the cycles of S_4_${\text{O}}_{6}^{2-}$ and S_2_${\text{O}}_{3}^{2-}$ are closely linked in both aqueous and sedimentary environments. In particular, the rapid consumption of S_4_${\text{O}}_{6}^{2-}$ in sediment experiments, which was also observed in marine environments, indicates a prevalent biological role for this intermediate. Moreover, the production of S_4_${\text{O}}_{6}^{2-}$ during thiosulfate consumption points to a potential source for low concentrations of S_4_${\text{O}}_{6}^{2-}$ in sediments, as S_2_${\text{O}}_{3}^{2-}$ consumption is significant throughout the sediment column in both freshwater and marine systems (Jørgensen, 1990a,b). Such loops within the oxidative sulfur cycle represent rate-determining processes that serve to retard the overall transformation of sulfide to sulfate.

## Conclusions

Consumption rates for intermediate sulfur species in a freshwater lake were measured in both aqueous and sedimentary incubation experiments. Concentration-normalized consumption rates were ${\text{S}}_{\text{colloidal}}^{0}>$ ${\text{S}}_{\text{x}}^{2-}>$ ${\text{SO}}_{3}^{2-}\approx$ S_4_${\text{O}}_{6}^{2-}>$ S_2_${\text{O}}_{3}^{2-}$ in sediment incubations and ${\text{S}}_{\text{colloidal}}^{0}>$ ${\text{SO}}_{3}^{2-}>$ ${\text{S}}_{\text{x}}^{2-}>$ S_2_${\text{O}}_{3}^{2-}>$ S_4_${\text{O}}_{6}^{2-}$ in aqueous incubation. In sediment slurry experiments, rapid consumption of H_2_S, ${\text{S}}_{\text{x}}^{2-}$, and ${\text{S}}_{\text{colloidal}}^{0}$ is likely due to reaction with reactive iron minerals in the anoxic, non-sulfidic sediment. Consumption of S_2_${\text{O}}_{3}^{2-}$, S_4_${\text{O}}_{6}^{2-}$, ${\text{SO}}_{3}^{2-}$ is likely primarily biological. With the exception of polysulfides, consumption of all other intermediates (${\text{S}}_{\text{colloidal}}^{0}$, S_2_${\text{O}}_{3}^{2-}$, S_4_${\text{O}}_{6}^{2-}$, ${\text{SO}}_{3}^{2-}$) was predominantly biological in aqueous incubations. These experiments provide the first measurement of the turnover of intermediate sulfur species in aqueous environments and of consumption rates for ${\text{S}}_{\text{x}}^{2-}$ and ${\text{S}}_{\text{colloidal}}^{0}$ in sediments. Moreover, they illustrate the biogeochemical complexity imparted by the production and consumption of these intermediates, which is hidden within “cryptic” sulfur cycling yet can be faster than that of either sulfide or sulfate. Internal cycles within intermediate species, for example between S_2_${\text{O}}_{3}^{2-}$ and S_4_${\text{O}}_{6}^{2-}$, add an additional layer of complexity and may slow the complete oxidation of sulfide within anoxic sediments and water columns.

## Author contributions

AF and AK designed the study, AF conducted the field and laboratory work, AF and AK wrote the manuscript.

### Conflict of interest statement

The authors declare that the research was conducted in the absence of any commercial or financial relationships that could be construed as a potential conflict of interest.

## References

[B1] Alam M. Pyne P. Mazumdar A. Peketi A. Ghosh W. (2013). Kinetic enrichment of 34S during proteobacterial thiosulfate oxidation and the conserved role of SoxB in S-S bond breaking. Appl. Environ. Microbiol. 79, 4455–4464. 10.1128/AEM.00956-13 23686269 PMC3697525

[B2] Bak F. Schuhmann A. Jansen K. (1993). Determination of tetrathionate and thiosulfate in natural samples and microbial cultures by a new, fast and sensitive ion chromatographic *technique*. FEMS Microbiol. Ecol. 12, 257–264. 10.1111/j.1574-6941.1993.tb00038.x

[B3] Barrett E. L. Clark M. A. (1987). Tetrathionate reduction and production of hydrogen sulfide from thiosulfate Microbiol. Rev. 51, 192–205. 3299028 10.1128/mr.51.2.192-205.1987 PMC373103

[B4] Beinart R. A. Gartman A. Sanders J. G. Luther G. W. Girguis P. R. (2015). The uptake and excretion of partially oxidized sulfur expands the repertoire of energy resources metabolized by hydrothermal vent symbioses. Proc. Biol. Sci. 282:20142811 10.1098/rspb.2014.2811 25876848 PMC4426611

[B5] Breier J. A. Toner B. M. Fakra S. C. Marcus M. A. White S. N. Thurnherr A. M. (2012). Sulfur, sulfides, oxides and organic matter aggregated in submarine hydrothermal plumes at 9°50°N East Pacific Rise. Geochim. Cosmochim. Acta 88, 216–236. 10.1016/j.gca.2012.04.003

[B6] Burdige D. J. Nealson K. N. (1986). Chemical and microbiological studies of sulfide-mediated manganese reduction. Geomicrobiol. J. 4, 361–387. 10.1080/01490458609385944

[B7] Canfield D. E. Stewart F. J. Thamdrup B. De Brabandere L. Dalsgaard T. Delong E. F.. (2010). A cryptic sulfur cycle in oxygen minimum zone waters off the Chilean coast. Science 330, 1375–1378. 10.1126/science.1196889 21071631

[B8] Chen K. Y. Morris C. J. (1972). Kinetics of oxidation of sulfide by oxygen. Environ. Sci. Tech. 6, 529–537. 10.1021/es60065a008

[B9] Chen K. Y. Gupta S. K. (1973). Formation of polysulfides in aqueous solution. Environ. Lett. 4, 187–200. 10.1080/00139307309436596 4686873

[B10] Childress J. J. Fisher C. R. Favuzzi A. Kochevar R. E. Sanders N. K. Alayse A. M. (1991). Sulfide-driven autotrophic balance in the bacterial symbiont-containing hydrothermal vent tubeworm, riftia pachyptila jones. Biol. Bull. 180, 135–153 10.2307/1542437 29303639

[B11] Childress J. J. Girguis P. (2011). The metabolic demands of endosymbiotic chemoautotrophic metabolism on host physiological capacities. J. Exp. Biol. 214, 312–325. 10.1242/jeb.049023 21177951

[B12] Cline J. D. (1969). Spectrophotometric determination of hydrogen sulfide in natural waters. Limnol. Oceanogr. 14, 454–458.

[B13] Cypionka H. Smock A. M. Bottcher M. E. (1998). A combined pathway of sulfur compound disproportionation in *Desutfovibrio desulfuricans*. FEMS Microbiol. Lett. 166, 181–186, 10.1111/j.1574-6968.1998.tb13888.x

[B14] Dahl C. (2008). Inorganic sulfur compounds as electron donors in purple sulfur bacteria. Sulfur Metab. Phototrophic Org. 289–317. 10.1007/978-1-4020-6863-8_15

[B15] Dinegar R. H. Smellie R. H. La Mer V. K. (1951). Kinetics of the acid decomposition of sodium thiosulfate in dilute solutions. J. Am. Chem. Soc. 73, 2050–2054. 10.1021/ja01149a043

[B16] dos Santos Afonso M. Stumm W. (1992). Reductive dissolution of iron(III) (hydr)oxides by hydrogen sulfide. Langmuir 8, 1671–1675. 10.1021/la00042a030

[B17] Eckert T. (2000). The Influence of Chemical Stratification in The Water Column on Sulfur and Iron Dynamics in Pore Waters and Sediments of Lake Kinneret, Israel. Masters Thesis, Universität Bayreuth.

[B18] Eckert W. Conrad R. (2007). Sulfide and methane evolution in the hypolimnion of a subtropical lake: a three-year study. Biogeochemistry 82, 67–76. 10.1007/s10533-006-9053-3

[B19] Eddie B. J. Hanson T. E. (2013). Chlorobaculum tepidum TLS displays a complex transcriptional response to sulfide addition. J. Bacteriol. 195, 399–408. 10.1128/JB.01342-12 23161024 PMC3553837

[B20] Elsgaard L. Jørgensen B. B. (1992). Anoxic transformations of radiolabelled hydrogen sulfide in marine and freshwater sediments. Geochim. Cosmochim. Acta 56, 2425–2435. 10.1016/0016-7037(92)90199-S

[B21] Findlay A. J. (2016). Microbial impact on polysulfide dynamics in the environment. FEMS Microbiol. Lett. 363:fnw103. 10.1093/femsle/fnw103 27190288

[B22] Findlay A. J. Gartman A. MacDonald D. J. Hansona T. E. Shawc T. J. Luther G. W. III (2014). Distribution and size fractionation of elemental sulfur in aqueous environments: the Chesapeake Bay and Mid-Atlantic Ridge. Geochim. Cosmochim. Acta 142, 334–348. 10.1016/j.gca.2014.07.032

[B23] Fisher C. R. Childress J. J. Arp A. J. Brooks J. M. Distel D. Favuzzi J. A. (1988). Physiology, morphology, and biochemical composition of Riftia pachyptila at Rose Garden in 1985. Deep-Sea Res. 35, 1745–1758. 10.1016/0198-0149(88)90047-7

[B25] Fossing H. Jørgensen B. B. (1990). Oxidation and reduction of radioloabeled inorganic sulfur compounds in estuarine sediment, Kysin Fjord, Denmark. Geochim. Cosmochim. Acta 54, 2731–2742. 10.1016/0016-7037(90)90008-9

[B26] Fossing H. Thode-Andersen S. Jørgensen B. (1992). Sulfur isotope exchange between 35 S-labeled inorganic sulfur compounds in anoxic marine sediments. Mar. Chem. 38, 117–132. 10.1016/0304-4203(92)90071-H

[B27] Frigaard N. U. Dahl C. (2008). Sulfur metabolism in phototrophic sulfur bacteria. Adv. Microb. Physiol. 54, 103–200. 10.1016/S0065-2911(08)00002-7 18929068

[B28] Fuseler K. Krekeler D. Sydow U. Cypionka H. (1996). A common pathway of sulfide oxidation by sulfate-reducing bacteria. FEMS Microbiol. Lett. 144, 129–134. 10.1111/j.1574-6968.1996.tb08518.x

[B29] Glombitza C. Adhikari R. R. Riedinger N. Gilhooly W. P. III. Hinrichs K. U. Inagaki F. (2016). Microbial sulfate reduction potential in coal-bearing sediments down to ~2.5 km below the seafloor off Shimokita Peninsula, Japan. Front. Microbiol. 7, 1–15. 10.3389/fmicb.2016.01576 27761134 PMC5051215

[B30] Griesbeck C. Hauska G. Schütz M. (2000). Biological sulfide oxidation: sulfide-quinone reductase (SQR), the primary reaction, in Recent Research Developments in Microbiology, Vol. 4, ed Pandalai S. G. (Trivadrum: Research Signpost), 179–203.

[B31] Gun J. Modestov A. D. Kamyshny A. Jr. Ryzkov D. Gitis V. Goifman A. (2004). Electrospray ionization mass spectrometric analysis of aqueous polysulfide solutions. Microchim Acta 146, 229–237. 10.1007/s00604-004-0179-5

[B32] Hansel C. M. Lentini C. J. Tang Y. Johnston D. T. Wankel S. D. Jardine P. M. (2015). Dominance of sulfur-fueled iron oxide reduction in low-sulfate freshwater sediments. ISME J. 9, 2400–2412. 10.1038/ismej.2015.50 25871933 PMC4611504

[B33] Henneke E. Luther G. W. De Lange G. J. Hoefs J. (1997). Sulphur speciation in anoxic hypersaline sediments from the eastern Mediterranean Sea. Geochim. Cosmochim. Acta 61, 307–321. 10.1016/S0016-7037(96)00355-9

[B34] Heunisch G. W. (1977). Stoichiometry of the reaction of sulfites with hydrogen sulfide ion. Inorg. Chem. 16, 1411–1413. 10.1021/ic50172a033

[B35] Holmkvist L. Ferdelman T. G. Jørgensen B. B. (2011). A cryptic sulfur cycle driven by iron in the methane zone of marine sediment (Aarhus Bay, Denmark). Geochim. Cosmochim. Acta 75, 3581–3599. 10.1016/j.gca.2011.03.033

[B36] Janek Y. A. (1933). Herstellung von Schwefelsolen. Kolloiden. 1, 31–32.

[B37] Janssen A. De Keizer A. Van Aelst A. Fokkink R. Yanglinga H. Lettingaa G. (1996). Surface characteristics and aggregation of microbiologically produced sulfur particles in relation to the process conditions. Colloids Surf. B. Biointerfaces 6, 115–129. 10.1016/0927-7765(95)01246-X

[B38] Jørgensen B. B. (1990a). The sulfur cycle of freshwater sediments: role of thiosulfate. Limnol. Oceanogr. 35, 1329–1342. 10.4319/lo.1990.35.6.1329

[B39] Jørgensen B. B. (1990b). A thiosulfate shunt in the sulfur cycle of marine sediments. Science 249, 152–154. 10.1126/science.249.4965.152 17836966

[B40] Jørgensen B. B. Bak F. (1991). Pathways and microbiology of thiosulfate transformations and sulfate reduction in a marine sediment (Kattegat, Denmark). App. Environ. Microbiol. 57, 847–856. 16348450 10.1128/aem.57.3.847-856.1991 PMC182805

[B41] Jørgensen B. B. Gijs Kuenen J. Cohen Y. (1979). Microbial transformations of sulfur compounds in a stratified lake (Solar Lake, Sinai). Limnol. Oceanogr. 24, 799–822. 10.4319/lo.1979.24.5.0799

[B42] Kamyshny A. (2009). Improved cyanolysis protocol for detection of zero-valent sulfur in natural aquatic systems. Limnol. Oceanogr. Methods 7, 442–448. 10.4319/lom.2009.7.442

[B43] Kamyshny A. Ekeltchik I. Gun J. Lev O. (2006). Method for the determination of inorganic polysulfide distribution in aquatic systems. Anal. Chem. 78, 2631–2639. 10.1021/ac051854a 16615773

[B44] Kamyshny A. Ferdelman T. G. (2010). Dynamics of zero-valent sulfur species including polysulfides at seep sites on intertidal sand flats (Wadden Sea, North Sea). Mar. Chem. 121, 17–26. 10.1016/j.marchem.2010.03.001

[B45] Kamyshny A. Jr. Goifman A. Gun J. Rizkov D. Lev O. (2004). Equilibrium distribution of polysulfide ions in aqueous solutions at 25 degrees C: a new approach for the study of polysulfides' equilibria. Environ. Sci. Technol. 38, 6633–6644. 10.1021/es049514e 15669322

[B46] Kamyshny A. Jr. Gun J. Rizkov D. Voitsekovski T. Lev O. (2007). Equilibrium distribution of polysulfide ions in aqueous solutions at different temperatures by rapid single phase derivatization. Environ. Sci. Technol. 41, 2395–2400. 10.1021/es062637+ 17438792

[B47] Kamyshny A. J. Druschel G. Mansaray Z. F. Farquhar J. (2014). Multiple sulfur isotopes fractionations associated with abiotic sulfur transformations in Yellowstone National Park geothermal springs. Geochem. Trans. 15:7. 10.1186/1467-4866-15-7 24959098 PMC4055273

[B48] Kamyshny A. Zerkle A. L. Mansaray Z. F. Ciglenečki I. Bura-Nakić E. Farquha J. (2011). Biogeochemical sulfur cycling in the water column of a shallow stratified sea-water lake: speciation and quadruple sulfur isotope composition. Mar. Chem. 127, 144–154. 10.1016/j.marchem.2011.09.001

[B49] Kleinjan W. E. de Keizer A. Janssen A. J. H. (2003). Biologically produced sulfur, in Elemental Sulfur and Sulfur-Rich Compounds I. Topics in Current Chemistry, Vol. 230, ed Steudel R. (Berlin; Heidelberg: Springer), 167–188. 10.1007/b12114

[B50] Kleinjan W. E. De Keizer A. Janssen A. J. H. (2005). Kinetics of the chemical oxidation of polysulfide anions in aqueous solution. Water Res. 39, 4093–4100. 10.1016/j.watres.2005.08.006 16213542

[B51] Knossow N. Blonder B. Eckert W. Turchyn A. V. Antler G. Kamyshny A. Jr. (2015). Annual sulfur cycle in a warm monomictic lake with sub-millimolar sulfate concentrations. Geochem. Trans. 16:7. 10.1186/s12932-015-0021-5 26140024 PMC4488043

[B52] Krämer M. Cypionka H. (1989). Sulfate formation via ATP sulfurylase in thiosulfate and sulfite-disproportionating bacteria. Arch. Microbiol. 151, 232–237. 10.1007/BF00413135

[B53] Li X. Taylor G. T. Astor Y. Scranton M. I. (2008). Relationship of sulfur speciation to hydrographic conditions and chemoautotrophic production in the Cariaco Basin. Mar. Chem. 112, 53–64. 10.1016/j.marchem.2008.06.002

[B54] Lichtschlag A. Kamyshny A. Ferdelman T. G. deBeer D. (2012). Intermediate sulfur oxidation state compounds in the euxinic surface sediments of the Dvurechenskii mud volcano (Black Sea). Geochim. Cosmochim. Acta 105, 130–145. 10.1016/j.gca.2012.11.025

[B55] Lie T. J. Godchaux W. Leadbetter E. R. (1999). Sulfonates as terminal electron acceptors for growth of sulfite-reducing bacteria (Desulfitobac- terium sp.) and sulfate-reducing bacteria: effects of inhibitors of sulfido- genesis. Appl. Environ. Microbiol. 65, 4611–4617. 10508097 10.1128/aem.65.10.4611-4617.1999 PMC91615

[B56] Luther G. W. III. (1987). Pyrite oxidation and reduction: molecular orbital theory considerations. Geochim. Cosmochim. Acta 51, 3193–3199. 10.1016/0016-7037(87)90127-X

[B57] Luther G. W. Findlay A. J. MacDonald D. J. Owings S. M. Hanson T. E. Beinart R. A.. (2011). Thermodynamics and kinetics of sulfide oxidation by oxygen: a look at inorganically controlled reactions and biologically mediated processes in the environment. Front. Microbiol. 2, 1–9. 10.3389/fmicb.2011.00062 21833317 PMC3153037

[B58] Luther G. W. III. Church T. M. Scudlark J. R. Cosman M. (1986). Inorganic and organic sulfur cycling in salt-marsh porewaters. Science 232, 746–749. 10.1126/science.232.4751.746 17769570

[B59] Ma S. Noble A. Butcher D. Trouwborst R. E. Luther G. W. III. (2006). Removal of H2S via an iron catalytic cycle and iron sulfide precipitation in the water column of dead end tributaries. Estuar. Coast. Shelf Sci. 70, 461–472. 10.1016/j.ecss.2006.06.033

[B60] Mason J. Kelly D. P. (1988). Thiosulfate oxidation by obligately heterotrophic bacteria. Microb. Ecol. 15, 123–134. 10.1007/BF02011707 24202996

[B61] Mills J. V. Antler G. Turchyn A. V. (2016). Geochemical evidence for cryptic sulfur cycling in salt marsh sediments. Earth Planet. Sci. Lett. 453, 23–32. 10.1016/j.epsl.2016.08.001

[B62] Newton G. L. Dorian R. Fahey R. C. (1981). Analysis of biological thiols: derivatization with monobromobimane and separation by reverse-phase high-performance liquid chromatography. Anal. Biochem. 114, 383–387. 10.1016/0003-2697(81)90498-X 7304929

[B63] O'Brien D. J. Birkner F. B. (1977). Kinetics of oxygenation of reduced sulfur species in aqueous solution. Environ. Sci. Technol. 11, 1114–1120. 10.1021/es60135a009

[B64] Podgorsek L. Imhoff J. F. (1999). Tetrathionate production by sulfur oxidizing bacteria and the role of tetrathionate in the sulfur cycle of Baltic Sea sediments. Aquat. Microb. Ecol. 17, 255–265. 10.3354/ame017255

[B65] Pyzik A. J. Sommer S. E. (1981). Sedimentary iron monosulfides: kinetics and mechanism of formation. Geochim. Cosmochim. Acta 45, 687–698. 10.1016/0016-7037(81)90042-9

[B66] Rickard D. Luther G. W. (2007). Chemistry of iron sulfides Chemical Reviews 107, 514–562. 10.1021/cr0503658 17261073

[B67] Rimmer A. Ostrovsky I. Yacobi Y. Z. (2008). Light availability for Chlorobium phaebacteroides development in Lake Kinneret. J. Plankton Res. 30, 765–776. 10.1093/plankt/fbn037

[B68] Rong L. Lim L. W. Takeuchi T. (2005). Determination of iodide and thiocyanate in seawater by liquid chromatography with poly(ethylene glycol) stationary phase. Chromatographia 61, 371–374. 10.1365/s10337-005-0501-3

[B69] Schippers A. Jørgensen B. B. (2002). Biogeochemistry of pyrite and iron sulfide oxidation in marine sediments. Geochim. Cosmochim. Acta 66, 85–92. 10.1016/S0016-7037(01)00745-1

[B70] Schwarzenbach G. Fischer A. (1960). Die Aciditat der Sulfane und die Zusammensetzung wasseriger Polysulfidlosungen. Helv. Chim. Acta 241, 1365–1390. 10.1002/hlca.19600430521

[B71] Sorokin D. Y. Teske A. Robertson L. A. Kuenen J. G. (1999). Anaerobic oxidation of thiosulfate to tetrathionate by obligately heterotrophic bacteria, belonging to the Pseudomonas stutzeri group. FEMS Microbiol. Ecol. 30, 113–123. 10.1111/j.1574-6941.1999.tb00640.x 10508936

[B72] Sorokin D. Y. Teske A. Robertson L. A. Kuenen J. G. (1996). Oxidation of sulfide and elemental sulfur to tetrathionate by chemoorganoheterotrophic bacteria. Microbiology 65, 5–9.

[B73] Steudel R. Göbel T. Holdt G. (1988). The molecular composition of hydrophilic sulfur sols prepared by acid decomposition of thiosulfate [1]. Z. Naturforsch. C. 43, 203–218. 10.1515/znb-1988-0212

[B74] Tuttle J. H. Jannasch H. W. (1972). Occurrence and types of thiobacillus-like bacteria in the sea. Limnol. Oceanogr. 17, 532–543.

[B75] Tuttle J. H. Jannasch H. W. (1973). Sulfide and thiosulfate oxidising bacteria in anoxic marine basins. Mar. Biol. 20, 64–70. 10.1007/BF00387676

[B76] Vainshtein M. B. Matrosov A. G. Baskunov V. P. Zyakun A. M. Ivanov M. V. (1980). Thiosulfate as an intermediate product of bacterial sulfate reduction. Mikrobiologiya 49, 855–858. 7207258

[B77] Vairavamurthy A. Zhou W. Eglington X. Manowitz B. (1994). Sulfo nates: a novel class of organic sulfur compounds in marine sediments: *Geoehimiea et Cosmochimica* Acta 58, 4681–4687. 10.1016/0016-7037(94)90200-3

[B78] van den Ende F. P. van Gemerden H. (1993). Sulfide oxidation under oxygen limitation by a thiobacillus thioparus isolated from a marine microbial mat. FEMS Microbiol. Ecol. 13, 69–77. 10.1111/j.1574-6941.1993.tb00052.x

[B79] van Gemerden H. (1986). Production of elemental sulfur by green and purple sulfur bacteria. Arch. Microbiol. 146, 52–56.

[B80] Yao W. Millero F. J. (1996). Oxidation of hydrogen sulfide by hydrous Fe(III) oxides in seawater. Mar. Chem. 52, 1–16. 10.1016/0304-4203(95)00072-0

[B81] Yücel M. Konovalov S. K. Moore T. S. Janzen C. P. Luther G. W. III (2010). Sulfur speciation in the upper Black Sea sediments. Chem. Geol. 269, 364–375. 10.1016/j.chemgeo.2009.10.010

[B82] Zerkle A. L. Kamyshny A. Kump L. R. James F. Daniel O. H. Michael A. A. (2010). Sulfur cycling in a stratified euxinic lake with moderately high sulfate: Constraints from quadruple S isotopes. Geochim. Cosmochim. Acta 74, 4953–4970. 10.1016/j.gca.2010.06.015

[B83] Zhang J. Millero F. J. (1991). The rate of sulfite oxidation in Seawater. 55, 677–685. 10.1016/0016-7037(91)90333-Z

[B84] Zhang J. Z. Millero F. J. (1993). The products from the oxidation of H2S in seawater. Geochem Cosmochim Acta 57, 1705–1718. 10.1016/0016-7037(93)90108-9

[B85] Zopfi J. Ferdelman T. G. Fossing H. (2004). Distribution and fate of sulfur intermediates - sulfite, tetrathionate, thiosulfate and elemental sulfur - in marine sediments. Spec. Pap. Geol. Soc. Am. 379, 17–34. 10.1130/0-8137-2379-5.97

[B86] Zöphel A. Kennedy M. C. Beinart H. Kroneck P. M. H. (1988). Investigations on microbial sulfur respiration 1. Activation and reduction of elemental sulfur un several strains of eubacteria. Arch. Microbiol. 150, 72–77. 10.1007/BF00409720

